# An integrated approach identifies new oncotargets in melanoma

**DOI:** 10.18632/oncotarget.23727

**Published:** 2017-12-15

**Authors:** Daniela Cecconi, Luca Dalle Carbonare, Antonio Mori, Samuele Cheri, Michela Deiana, Jessica Brandi, Vincenzo Degaetano, Valentina Masiero, Giulio Innamorati, Monica Mottes, Giovanni Malerba, Maria Teresa Valenti

**Affiliations:** ^1^ Department of Biotechnology, Mass Spectrometry and Proteomics Lab, University of Verona, 37134 Verona, Italy; ^2^ Department of Medicine, Internal Medicine, Section D, University of Verona, 37134 Verona, Italy; ^3^ Department of Neuroscience, Biomedicine and Movement Sciences, University of Verona, 37134 Verona, Italy

**Keywords:** melanoma, oncotarget, ascorbic acid, gene expression, proteomics

## Abstract

Melanoma is an aggressive skin cancer; an early detection of the primary tumor may improve its prognosis. Despite many genes have been shown to be involved in melanoma, the full framework of melanoma transformation has not been completely explored. The characterization of pathways involved in tumor restraint in *in vitro* models may help to identify oncotarget genes. We therefore aimed to probe novel oncotargets through an integrated approach involving proteomic, gene expression and bioinformatic analysis

We investigated molecular modulations in melanoma cells treated with ascorbic acid, which is known to inhibit cancer growth at high concentrations. For this purpose a proteomic approach was applied. A deeper insight into ascorbic acid anticancer activity was achieved; the discovery of deregulated processes suggested further biomarkers. In addition, we evaluated the expression of identified genes as well as the migration ability in several melanoma cell lines.

Data obtained by a multidisciplinary approach demonstrated the involvement of Enolase 1 (ENO1), Parkinsonism-associated deglycase (PARK7), Prostaglansin E synthase 3 (PTGES3), Nucleophosmin (NPM1), Stathmin 1 (STMN1) genes in cell transformation and identified Single stranded DNA binding protein 1 (SSBP1) as a possible onco-suppressor in melanoma cancer.

## INTRODUCTION

Malignant melanoma (MM) has been considered a rare cancer for a long time. However, in the last years incidence of MM has increased considerably in consequence of lifestyle and environmental changes. The mortality rate for MM is very high as it is highly invasive and also genetically resistant to chemotherapeutic treatments. The use of cytokines has been considered an effective tool against melanoma thanks to their ability to stimulate the immune system. In fact, an immunological pathogenetic mechanism has been suggested to be involved in the natural regression occurring in the primary tumor. Recently, the opportunity to characterize this cancer at the molecular level has improved therapeutical strategies, yet biased by chemoresistance. The molecular aspects underlying chemoresistance acquisition are unknown. Many studies, performed in animal models and in primary tumors, shed light on the complex genomic background involved in metastatic progression of MM; it has also been reported that mutation rate and gene modulation in melanoma are higher than in other solid malignancies [1, 2]. With the aims of: i) identifying oncotargets and ii) studying proteome variations involved in an antitumor activity, we investigated the pathways modulated by ascorbic acid (AsA) in melanoma cells [3]. It has been actually demonstrated *in vitro* that AsA reduces the malignant potential [4, 5] in a murine melanoma model [6] as well as in human melanoma [3].

It should first be emphasized that ascorbic acid acts as a co-factor in many biological reactions and is an essential water-soluble vitamin with antioxidant properties. Recent pharmacokinetics studies demonstrated that intravenously administered AsA reaches high plasma levels leading to tumor cytotoxicity. It is important to note that AsA affects tumor cells selectively. Antioxidant levels reduction [7–9] as well as an increased glycolytic metabolism leading to DHA intracellular uptake [8] plus a higher sensitivity to H_2_O_2_, represent useful selective markers in cancer cells [10]. In our study, we analyzed proteome modulation in melanoma cells treated with AsA; gene expression levels for the identified proteins were then investigated as well as the migratory ability in several melanoma cells.

## RESULTS

### Proteomic profiling of melanoma cancer cells treated with AsA

**Figure 1:**
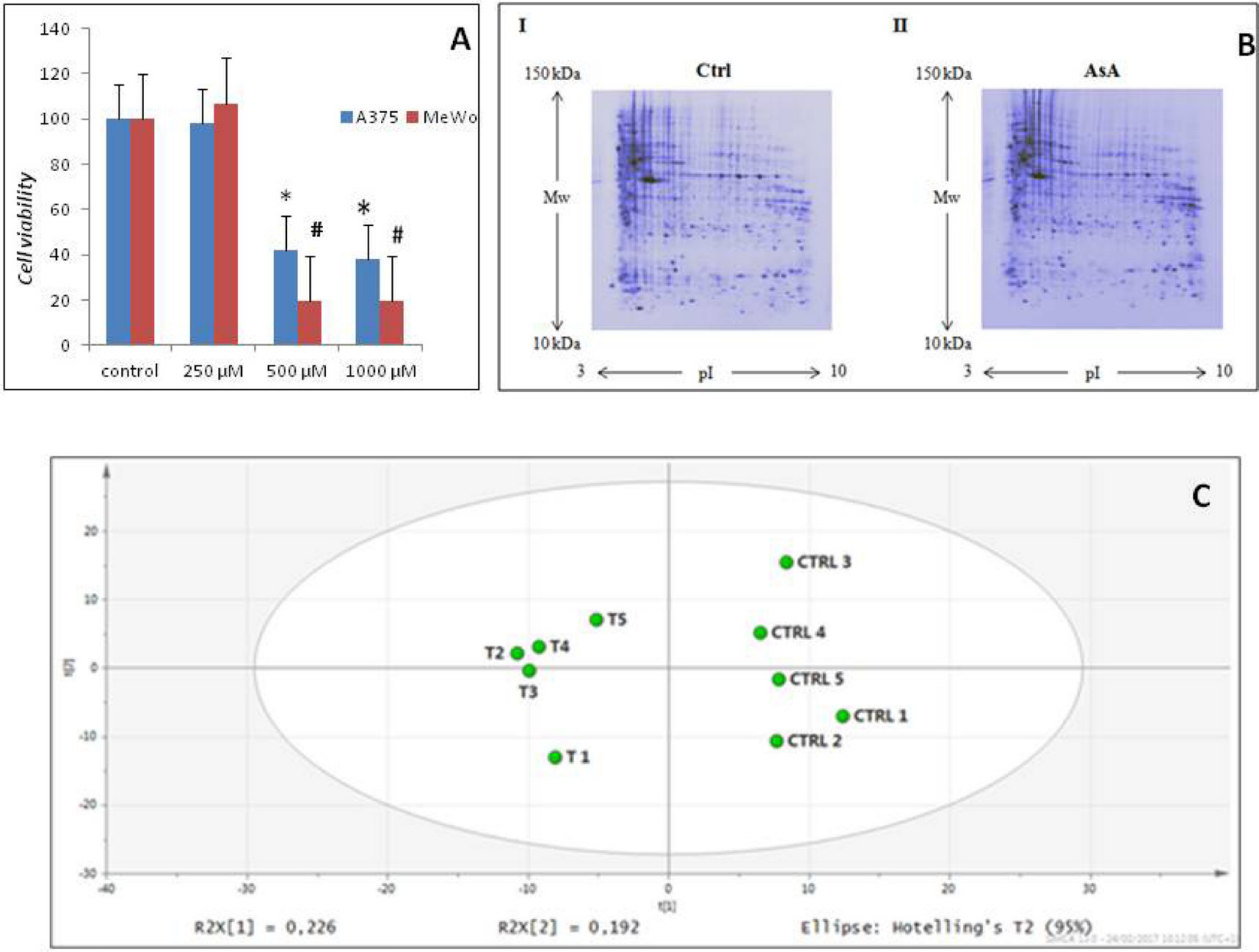
AsA treatment effects in melanoma cells (**A**) After treating A375 and Mewo cells with AsA at 500 and 1000 μM concentrations, a statistically significant dose-dependent reduction of cell viability was observed for both cell lines (^*^p < 0.05; ^#^*p* < 0.01). (**B**) Representative two-dimensional gel electrophoresis images obtained using a) control and b) AsA treated MeWo cells. (**C**) Principal component analysis of proteome patterns. The two principal components PC1 and PC2 were 22,6% and 19,2%, respectively. The PCA scatterplot clearly clustered the individual proteome maps into two distinct groups corresponding to the two experimental groups considered (controls and AsA treated cells).

In order to evaluate protein modulations by a molecule known to affect melanoma cells, we treated the MeWo and A375 cell lines with high concentrations of Ascorbic Acid. Our data showed a statistically significant dose-dependent reduction of cell viability in both cell lines at the highest concentrations used, i.e. 500 and 1000 μM, (Figure 1A).

On the basis of cell viability data, we chose to analyze the total proteome of MeWo cells before and after treatment with 500 μM AsA for 48 h, which was shown to inhibit cancer growth. An average of 371 (± 3%) protein spots with an apparent molecular mass between 10 and 200 kDa and a pI between 3 and 10 were detected by 2-DE. Representative two-dimensional maps of the two conditions are reported in Figure 1B. Multivariate statistical analysis was applied to the protein patterns in order to identify protein species responsible for correlated variations and to investigate intra- and inter-group relationships. PCA indicated two distinct groups of maps from the two experimental conditions, treated and untreated cells (Figure 1C). The strongest separation was observed in the first and second PCs, which accounted for most of the variation in the data sets, i.e. 22.6% and 19.2% respectively. These results indicate that natural variation in the proteomes is sufficient to allow discrimination between different groups, increasing the confidence that potential disease biomarkers and oncotargets can be found in this dataset.

Supported by multivariate and univariate analyses, a total of 34 spots from the 2-DE analysis were identified as significantly differentially regulated in control and AsA treated MeWo cells, respectively. All the modulated spots were successfully picked up and 29 protein species were identified by nano-HPLC-Chip ion trap MS/MS (Table 1). In Supplementary Figure 1, the identified protein species are marked with the numbers corresponding to the standard spot number (SSP) appearing in Table 1.

**Table 1:** Proteins identified by nano-HPLC-Chip ion trap MS/MS

Protein name	Gene name	Spot no. ^a)^ (Supplementary Figure 1)	NCBI acc. #	No. peptides identified	Mascot score	Mr. (Da) exp./theor.	pI exp./theor.	Seq. Coverage (%)	Fold change ^b)^	*p* (corr)	*p* value (< 0.01)
***Apoptosis:***											
10 kDa heat shock protein, mitochondrial	HSPE1	9004	gi|4504523	18	443	10000/10925	8.8/8.89	76	2.07	0.879	0.0016
adenylate kinase 2, mitochondrial	AK2	8202	gi|4502013	5	206	29000/26745	7.8/7.67	21	1.59	0.831	0.0043
Co-Chaperone P23	PTGES3	102	gi|9257073	4	112	20000/15216	4.5/5.09	36	−10	−0.774	0.0056
cyclophilin A	PPIA	8103	gi|1633054	9	270	17500/18154	7.8/7.82	35	1.54	0.885	0.0001
manganese superoxide dismutase	SOD2	7101	gi|62738405	7	214	22000/21864	7.3/6.86	27	1.64	0.909	0.0007
MICOS complex subunit MIC19	CHCHD3	9102	gi|8923390	3	135	27000/26491	8.4/8.48	8	1.82	0.884	0.0007
mitochondrial single-stranded DNA binding protein	SSBP1	8002	gi|2624694	5	163	15500/15186	7.8/8.23	36	1.77	−0.809	0.0002
profilin-1	PFN1	9002	gi|4826898	4	207	15000/15258	8.4/8.44	37	1.41	0.804	0.0089
profilin-1	PFN1	9001	gi|4826898	7	193	15000/15258	8.4/8.44	49	1.96	0.978	0.0000
protein DJ-1	PARK7	4108	gi|31543380	6	166	25000/20092	5.9/6.33	33	−1.56	−0.799	0.0038
voltage-dependent anion-selective channel protein 1	VDAC1	8307	gi|4507879	18	455	35000/30896	8.0/8.62	43	1.79	0.915	0.0004
***Cell migration, invasion, and metastasis:***											
alpha-enolase	ENO1	5610	gi|4503571	20	641	50000/47566	6.8/7.01	37	−1.54	−0.904	0.0004
alpha-enolase	ENO1	5608	gi|4503571	13	492	50000/47566	6.7/7.01	33	−1.54	−0.904	0.0002
B23 nucleophosmin	NPM1	1406	gi|825671	6	294	36000/31132	4.7/4.71	25	−1.72	/	0.0021
heat shock protein beta-1	HSPB1	5201	gi|4504517	12	208	28000/22840	6.2/5.98	33	1.31	0.869	0.0004
nuclear corepressor KAP-1	NCOR1	902	gi|1699027	2	79	90000/90682	4.6/5.52	3	−6.25	−0.810	0.0082
phosphatidylethanolamine-binding protein 1	PEBP1	7107	gi|4505621	3	197	21000/21186	7.6/7.01	22	1.39	0.895	0.0012
transgelin-2	TAGLN2	9101	gi|4507357	10	288	21000/22605	8.4/8.41	32	1.30	0.878	0.0024
***Oxidative stress:***											
albumin	ALB	4708	gi|119626083	4	110	60000/58614	5.8/6.66	6	1.87	0.832	0.0018
ATP synthase subunit d, mitochondrial	ATP5H	2103	gi|5453559	4	143	21000/18551	5.2/5.21	16	1.55	0.900	0.0002
malate dehydrogenase	MDH2	9307	gi|2906146	9	454	36000/36077	9.0/8.92	28	2.95	0.786	0.0085
peroxiredoxin-1	PRDX1	8107	gi|4505591	13	379	23000/22096	8.0/8.27	42	1.42	0.865	0.0030
***mRNA processing and translation:***											
40S ribosomal protein S21	RPS21	7004	gi|4506699	2	93	10000/9248	7.6/8.68	33	1.61	0.927	0.0003
elongation factor Tu	EFTUD	6501	gi|704416	9	294	49000/49935	6.9/7.7	22	1.58	0.814	0.0078
heterogeneous nuclear ribonucleoproteins A2/B1	HNRPA2B1	9312	gi|4504447	4	233	35000/35984	8.2/8.67	14	2.33	/	0.0000
heterogeneous nuclear ribonucleoproteins A2/B1	HNRPA2B1	9313	gi|4504447	4	140	35000/35984	8.6/8.67	14	2.54	0.869	0.0018
heterogeneous nuclear ribonucleoproteins A2/B1	HNRPA2B1	8306	gi|4504447	4	250	33000/36055	7.9/8.67	14	2.59	0.868	0.0014
***Cytoskeleton organization:***											
alpha-tubulin	TUBA	2707	gi|37492	5	186	55000/50978	5.2/5.02	14	−1.33	−0.837	0.0057
stathmin 1	STMN1	4101	gi|197692339	6	202	17500/17320	5.7/5.76	29	−1.49	−0.813	0.0040

^a)^ Spot numbers refer to those in Supplementary Figure 1 ^b)^ Fold change: the expression ratios between the means of spots value (% vol) of control and AsA treated MeWo cells resulting from digital image analysis. – Indicates decreased protein intensity.

### Gene ontology and protein interactions analysis

**Figure 2:**
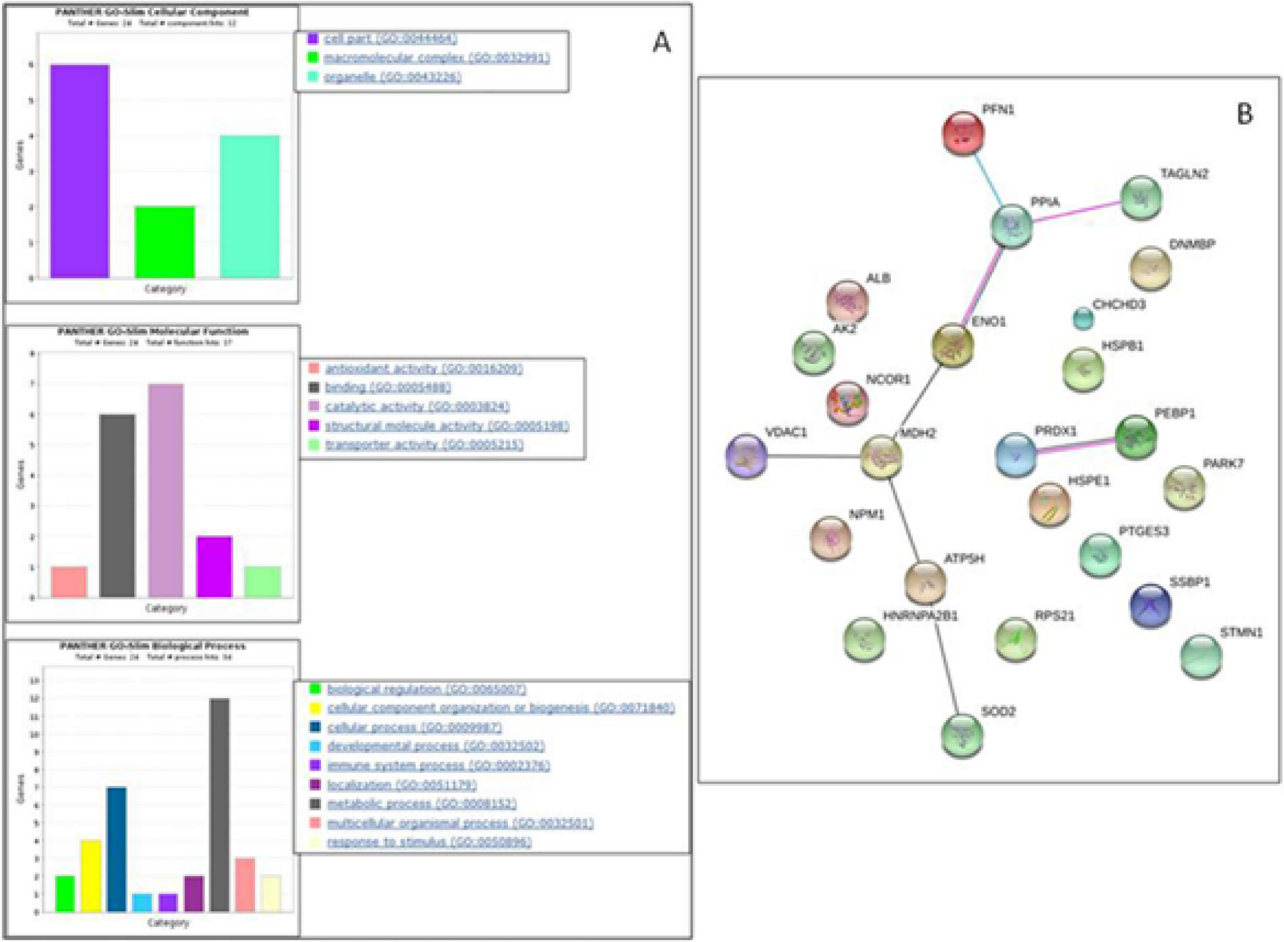
PANTHER functional classification of differentially expressed proteins between MeWo controls and MeWo cells treated with AsA (**A**) Histograms showing classification according to cellular component, molecular function, and biological process. (**B**) Schematic view of known and predicted protein interactions according to the STRING database (v. 10). Only interactions with the medium confidence score (0.400) are shown. Interactions include physical and functional associations, showing the evidence view. The lines indicate co-expression (grey), experimental (pink) or database (cyan) evidences.

By bioinformatic analyses, we found that the cellular components mostly over-represented in AsA-treated cells were cell part (25%), organelle (16%) and macromolecular complex (8%). As for the molecular function category, we found the catalytic activity (29%), binding (25%), and structural molecule activity (8%) among the most prominent significant GO enriched terms. In addition, the GO enrichment analysis suggested that proteins modulated by AsA are mainly involved in metabolic (50%) and cellular processes (29%), as well as in cellular components organization or biogenesis (17%) (Figure 2A). By STRING analysis we identified a core network of AsA-modulated proteins mainly involved in energetic metabolism, structure and folding (ATP5B, ENO1, MDH2, PFN1, PPIA, SOD2, TAGLN2 and VDAC1) suggesting a key role of AsA in these processes (Figure 2B).

### Protein-protein functional association in apoptosis and metastasis

**Figure 3:**
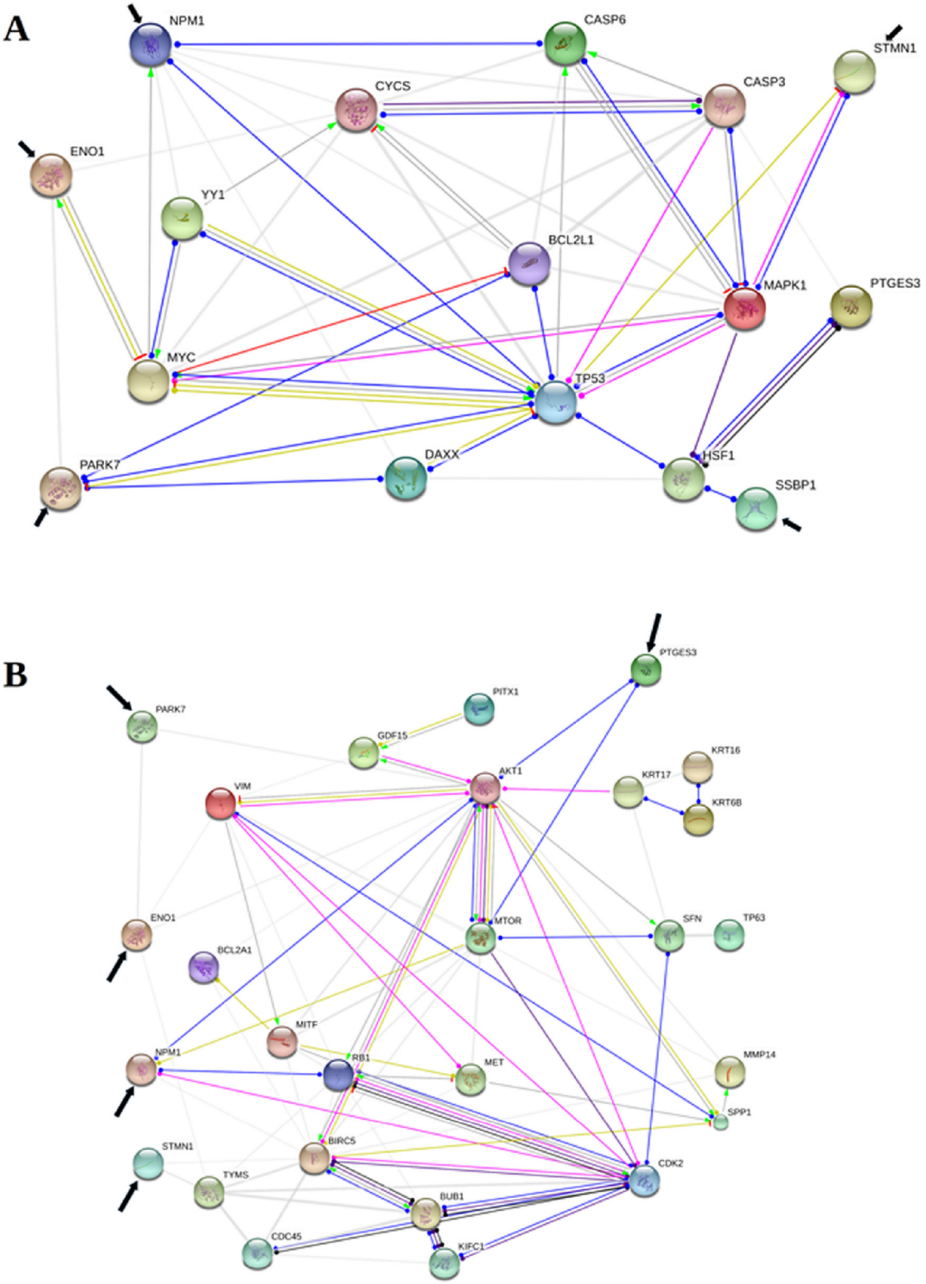
Protein-protein functional associations involved in apoptosis and mestastasis The figure shows a schematic visualization of the action types and action effects concerning ENO1, PARK7, PTGES3, NPM1, SSBP1, STMN1 (pointed by black arrow) among themselves and other proteins, known and predicted, to be involved in apoptosis (Protein-Protein Interaction -PPI- enrichment *p*-value < 0,01) (**A**) and metastasis (PPI enrichment *p*-value < 0,01) (**B**), according to the STRING v10.5 (only interactions with confidence score >= 0.400 are shown). Line colours and shapes indicate reaction (black), binding (blu), catalysis (indigo), transcriptional regulation (green mint), activation (light green), post-translational modification (fuchsia), inhibition (red), positive effect (arrowhead), negative effect bar), and unspecified effect (dot). Finally, the thickness of grey lines indicates the strength of data support.

The reconstruction by STRING of functional association in apoptosis showed, as eye-catching interaction, that PARK7, NPM1 and STMN1 were functionally associated with TP53; ENO1 and NPM1 with MYC; PTGES3 and SSBP1 with HSF1. PARK7 was also associated with BCL2L1 and DAXX; NPM1 with CASP6, and STMN1 with MAPK1. Furthermore the results showed interactions between PARK7 and ENO1; NPM1 and BCL2L1, DAXX, CASP3, CYC6, YY1; finally, ENO1 and CYCS, albeit with a lower strenght (Figure 3A; Supplementary Tables 1 and 2).

As for metastasis, NPM1 and PTGES3 were associated with MTOR and AKT1. Besides, NPM1 interacted with CDK2 and RB1. A lower strength was found in the association between PARK7 and ENO1 with AKT1; STMN1 with TYMS (Figure 3B; Supplementary Tables 3 and 4).

### Gene expression modulation and migration ability in AsA treated melanoma cells

We chose to investigate ENO1, PTGES3, NPM1, PARK7, STMN1 and SSBP1 gene expression. In fact, it has been reported that the above genes show increased expression in aggressive tumors, but data related to their role in melanoma are either scanty or still lacking.

**Figure 4:**
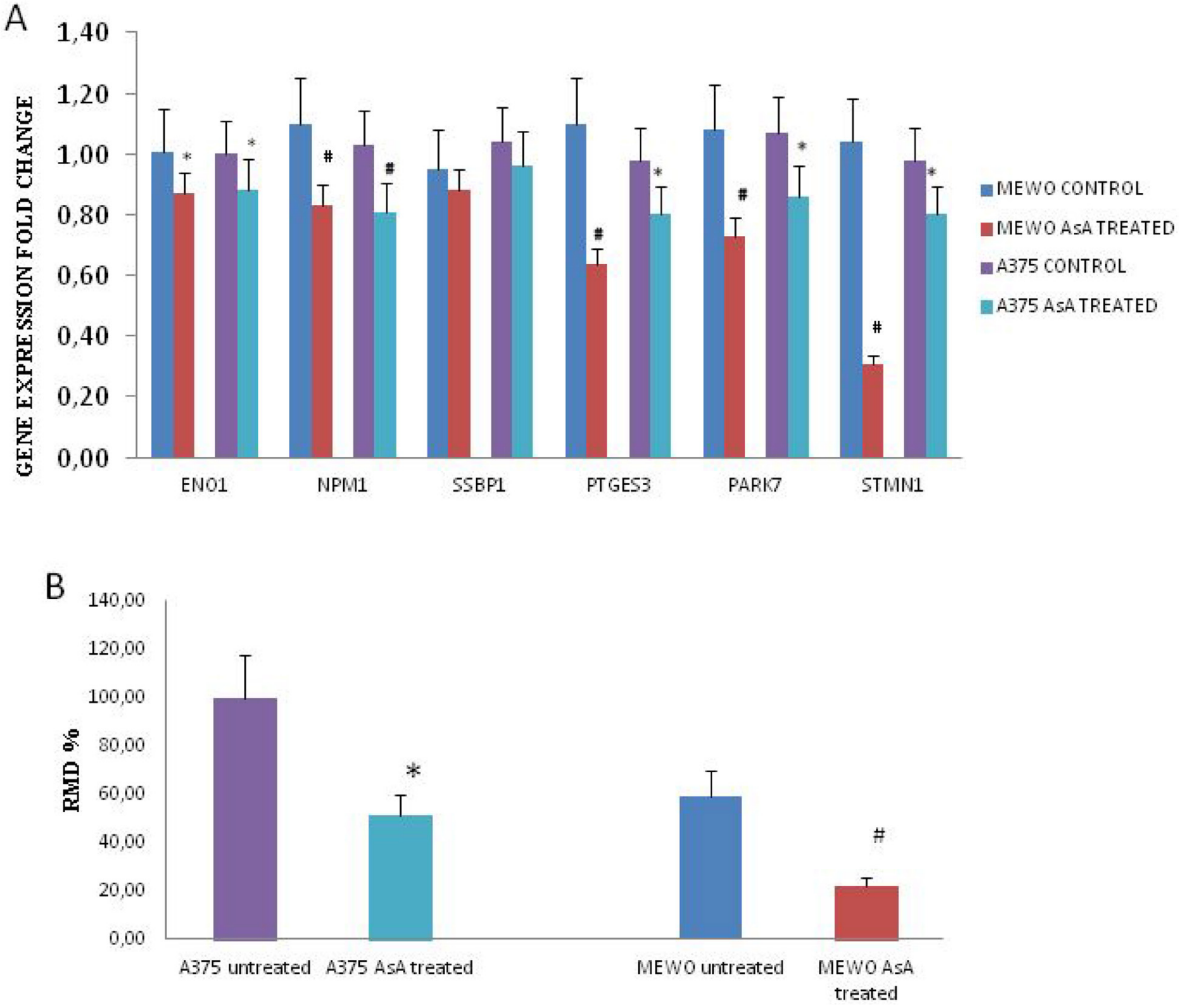
Gene expression modulation and migration ability in AsA treated melanoma cells (**A**) Gene expression profiles in untreated versus AsA treated Mewo and A375 cells. ^*^indicates significant values (*p* < 0.05) for Mewo cells; §indicates significant values (*p* < 0.05) for A375 cells. (**B**) The migration ability (expressed as RMD: Rate Migration Distance ) was significantly lower in both melanoma cell lines treated with AsA (^*^p < 0.05; ^#^*p* < 0.01).

Therefore, gene expression was monitored in cells treated with 500 μM AsA. Real time RT-PCR results showed that ENO1, PTGEs3/p23, NPM1, PARK7/DJ1 and STMN1/p18 genes expression was lower in AsA treated cells in both cell lines compared to controls (^*^p < 0.05; ^#^*p* < 0.01). No difference was observed when we analyzed SBBP1 gene expression in both cell lines. Figure 4A. In addition, both AsA treated cell lines showed a lower migration ability compared to untreated cells Figure 4B. Likewise gene expression and migration ability were evaluated in cells treated with A2P. As shown in Supplementary Figure 2 we found similar results even if A2P treatment affected cell viability also at a lower concentration

### Gene expression analyses and migration ability

**Figure 5:**
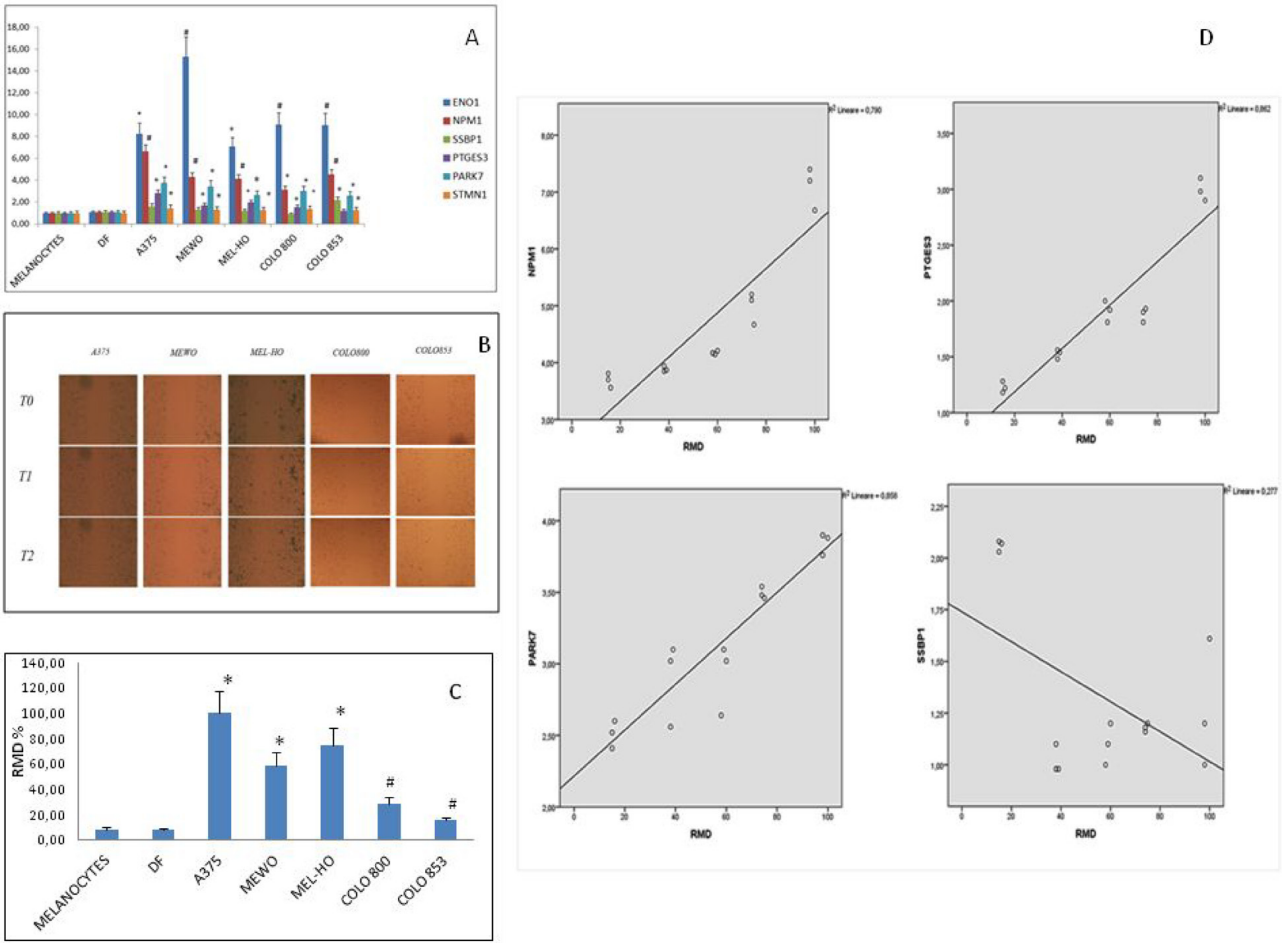
Gene expression analyses and migration ability evaluation in normal (fibroblasts and melanocytes) and melanoma cells (**A**) Stastically significant expression levels are indicated for each gene with ^*^p < 0.05 and ^#^*p* < 0.01, respectively. (**B**).Scratch test for migration ability evaluation (**C**) Relative Migration Distance values: ^*^p < 0.05), ^#^*p* < 0.01). (**D**) Correlation between gene expression levels and migration ability in melanoma cell lines. A positive correlation was found for NMP1, PTGS3 and PARK7, while a negative correlation was found for SSBP1.

In order to evaluate the involvement of the above genes in melanoma malignancy, we analyzed their expression levels in cancer and normal cells (melanocytes and fibroblasts); we also investigated the relationship between gene expression levels and migratory ability. We observed that ENO1, PARK7, NPM1 and STMN1 genes expression levels were higher in melanoma cells than in melanocytes and in normal fibroblasts (Figure 5A). In particular, *ENO1* and NMP1 expression levels were higher in all melanoma lines; PTGS3 expression was higher in all cell lines except for Colo853 melanoma cells. *SSBP1* instead was higher only in Colo853, compared to melanocytes and normal fibroblasts. Migration ability, evaluated by scratch test (Figure 5B) and expressed as relative migration distance (RMD) (Figure 4C), correlated with gene expression. Notably a positive correlation between NMP1, PTGS3 and PARK7 genes expression levels and migratory ability was found; conversely, a negative correlation was found between SSBP1 expression levels and cells migratory ability. (Figure 5D).

To confirm the relation between gene expression and migration ability we knocked down NMP1and PARK7 in A375 and Mewo cell lines by applying NMP1 and PARK7 specific shRNA sequences, respectively. In NMP1 and PARK 7 knock-down cells the expression of the above genes was reduced as well as migration ability, compared to A375 and Mewo WT cells (Supplementary Figure 3).

### Analysis of SKCM data from cancer genome atlas database

**Figure 6:**
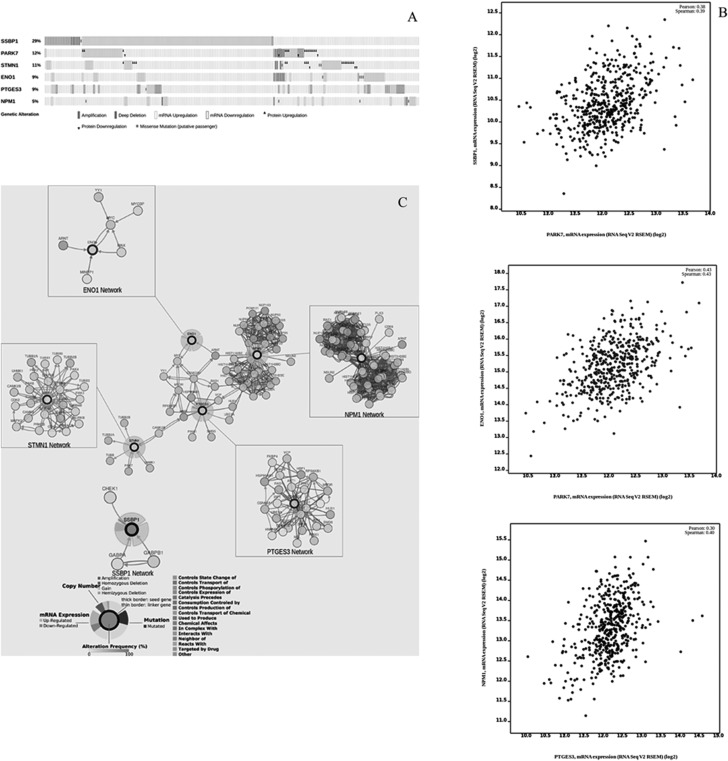
Genes involved in SKCM (**A**) Graphical summary of genomic alterations in *ENO1*, *PTGES3*, *NPM1*, *PARK7*, *STMN1* and *SSBP1* across the 479 SKCM samples from TCGA. Globally, the six queried genes are altered in 229/479 SKCM samples (47.8%). Row: queried gene and corresponding percentage. Bar: tumour sample. (**B**) Positive correlation data found for genes coexpression (**C**) Network analysis. Overall view of *ENO1*, *NPM1*, *PTGES3*, *STMN1* network interconnections. *SSPB1* network does not show any interconnection with the above network. No network was reported for PARK7 in SKCM. Each gene pathway is also represented individually. Nodes represent queried genes (thick border) and nearest neighbour-genes, which are shown with color-coded alteration frequency in SKCM. Edges are color-coded according to the different interaction types.

On the basis of the proteomic analysis results and in a translational-medicine perspective, we applied a bioinformatic approach to query about the corresponding genes and their involvement in Skin Cutaneous Melanoma (SKCM). For this purpose, we used the cBio Genomic Cancer Portal [11, 12] which allows to analyze the multidimensional harmonized cancer genomic datasets (included Cancer Genome Atlas database, TCGA) at the gene level. Specific biological events (e.g., mRNA expression and protein abundance changes) can be detected, networks based on biological pathways which involve genes of interest can be generated and epidemiological information (e.g. survival analysis) can be retrieved. The results showed that all six genes were globally altered in 229 of 479 samples queried (47,8%) (Figure 6A). Among them, *SSBP1* gene was the most frequently altered (141/479 = 29%), followed by *PARK7* (56/479), *STMN1* (51/479), *PTGES3* (44/479), *ENO1* (41/479) and, finally, *NPM1* (25/479). The alterations affecting them tended to take place concomitantly (Supplementary Table 5). Relatively to gene expression, the enrichment analysis showed a statistically significant over-expression of *SSBP1* (*p*-value = 1.09e-33; *Q*-value = 1.33e-29), *PARK7* (*p*-value = 8.16e-8; Q-value = 8.35e-7), and *ENO1* (*p*-value = 0.041; *Q*-value = 0.18). Notably, *SSBP1* resulted to be the first statistically significant within the gene list (12,192 entries of 20,532 mRNA) with altered mRNA expression in SKCM. When gene co-expression was queried, a positive correlation was found between *PARK7* and *ENO1* (Pearson correlation test: 0.43; *p*-value < 2.2e-16), (*PARK7* and *SSBP1* (Pearson correlation test: 0.38; *p*-value < 2.2e-16), *PTGES3* and *NPM1* (Pearson correlation test: 0.30; *p*-value = 2.463e-11) (Figure 6B). As for the enrichment analysis of protein expression, data are available only for *STMN1* and *PARK7* encoded proteins (235 entries). Stathmin 1 showed a statistically significant (*p*-value = 1.069e-4; *Q*-value = 5.024e-3) over-expression compared to the unaltered group; Deglycase DJ-1 tended to be slightly under-expressed, although the result was not statistically significant (*p*-value = 0.783; *Q*-value = 0.876). No result was found for the other gene products. No statistically significant correlation was found for concurrent protein expression.

### Network analysis in SKCM

In order to unearth the network complexity involving the investigated genes and their partners relevant to SKCM, we queried the Cbio Portal Network Analysis. The cBio Portal generated a network showing that *ENO1, PTGES3, NPM1* and *STMN1* are connected in this tumor (Figure 6C). *PARK7* and *SSBP1,* on the contrary, showed neither mutual connections nor any connection with the previously mentioned genes network

## DISCUSSION

Melanoma incidence has been increasing in recent times and the prognosis for metastatic melanoma is poor. An early detection of skin lesions and surgical treatment before cells dissemination represent successful therapeutic approaches; after metastases spreading, instead, effective tools against melanoma are limited. Despite initial positive responses, cells subsequently become resistant to apoptosis and chemotherapy. Thus the discovery of new oncotargets is essential in order to develop more effective anticancer agents. It should also be noted that oncotargets may be elicited by molecular changes concerning programmed cell death (apoptosis), proliferation, production of new blood vessels (angiogenesis), drug resistance, and potential of cells to initiate secondary tumors (metastasis) [13].

Here we consider a few oncotargets prompted by proteomic analysis, which were also supported and validated by gene expression and bioinformatic analyses. The proteomic results indicate that ascorbic acid down-modulates proteins involved in cell migration and invasiveness. Among these, the most strongly repressed protein is co-chaperone P23 (PTGES3, −10). Interestingly p23 is degraded during apoptosis induced by several stimuli, including Fas and TNFα-receptor activation [14]. Furthermore, it has been demonstrated that increased p23 expression may confer a more aggressive phenotype to cancer cells, promoting drug resistance, motility and metastasis [15]. PTGES3 gene is not only involved in the prostaglandin biosynthetic process (GO:0001516), but also in telomere maintenance (GO:0000723), signal transduction (GO:0007165), and xenobiotic metabolic process (GO:0006805). P23/TEBP, its product, takes part in cellular responses to stress (such as activation of Heat shock factor 1, HSF1) (GO:1900034), and is a co-chaperone of Heat Shock Protein 90 (HSP90). Precisely, P23/TEBP addresses the recruitment of prolyl hydroxylase domain protein 2 (EGLN1/PHD2) to the HSP90 pathway by facilitating HIF alpha proteins hydroxylation via interaction with EGLN1/PHD2. *HSF1* is alterated in 22% of samples (102/479, mainly mRNA over-expression) and encodes a transcription factor induced after heat shock stress to regulate lifespan. The strong down-regulation of co-chaperone P23 (−10) following ascorbic acid treatment of MeWo cells and the positive correlation between gene expression and migratory ability confirm that this protein is an important oncotarget on which searches for new drugs against melanoma can be addressed.

We also found that nucleophosmin production is down-regulated by AsA treatment (NPM1, −1,72); we have demonstrated a positive correlation between NPM1 gene expression and migratory ability, confirmed by RNA interference assay, as well. This protein may also represent a therapeutic target. Interestingly, it has been shown recently that nucleophosmin expression correlates with the migration and invasiveness of colon cancer cells [16]. In melanoma the over-expression of NPM1 has already been confirmed; it has also been reported that this protein undergoes hyperphosphorylation [17]. Nucleophosmin results to be engaged in various processes such as chromosome maintenance (i.e., nucleosome assembly and telomerase maintenance), DNA repair (GO:0006281), DNA damage response via p53 (GO:0006977), intracellular protein (GO:0006886) and nucleocytoplasmic (GO:0006913) transport, response to stress (GO:0006950), cell aging (GO:0007569), and HIF1 alpha transcription factor network. *NPM1* gene expression is controlled by *ARNT* (24% of alterations reported, mainly gene over-expression, in the TCGA SKCM samples), which encodes a co-factor for transcriptional regulation by hypoxia-inducible factor 1.

We also found DJ-1 protein (PARK7, −1.56) to be down-regulated after treatment with AsA. In addition we observed also a strong positive correlation between PARK 7 gene expression and migratory ability in melanoma cell line. This relation was confirmed by RNA interference assay as PARK7 knockdown reduced the migration ability. PARK7 encodes Deglycase DJ-1, a member of the peptidase C56 family, that is engaged in various processes such as negative regulation of TRAIL-activated apoptotic signaling pathway (GO:1903122), protein stabilization and physiology (GO:0050821), cadherin binding involved in cell-cell adhesion (GO:0098641), maintenance of correct mitochondrial morphology and function (GO:0007005) as well as autophagy of dysfunctional mitochondria [18]. It also acts as p53 inhibitor, and by inducing the vHL/HIF1 and PI3K/AKT/mTOR it promotes invasiveness, metastasis and chemotherapy resistance [19]. Furthermore, DJ-1 plays a fundamental role in counteracting cell death due to oxidative stress (GO:0034599) since it functions as an oxidative stress sensor and redox-sensitive chaperone and protease.

Alpha enolase, ENO1 gene product, was found to be down-regulated by ascorbic acid. It acts on the PI3K/AKT signal and correlates with cell invasiveness in various cancers [20]. Interestingly, ENO1 promotes invasion and metastasis formation also by acting as a plasminogen receptor [21]. The alpha enolase/MBP1 protein takes part in growth control, hypoxia tolerance processes (it is related to the HIF-1 signaling pathway). Moreover, MBP1 is involved in cell-cell adhesion (GO:0098609), cadherin binding involved in cell-cell adhesion (GO:0098641). Noteworthy, *ENO1* and *MYC* mutually control themselves for mRNA expression. MYC is an important regulator of cell cycle progression, apoptosis and cellular transformation. In the TCGA database, it is altered in 15% (69/479, mainly mRNA over-expression) SKCM samples. Interestingly, we found that two protein species of alpha-enolase are down-regulated in MeWo cells after ascorbic acid treatment (both −1.54), confirming a key role of this protein as oncotarget in melanoma.

STMN1, a member of the stathmin gene family, encodes the cytosolic phosphoprotein Stathmin 1, which plays a role in the regulation of microtubule organization. Furthermore, stathmin is involved in cytoskeletal signaling and G protein–coupled receptors pathway. STMN1 network is connected to *PTGES3* via the Ca(2+)/calmodulin-dependent protein kinase CAMK2B, which belongs to the serine/threonine protein kinase family and plays a role in different neuronal processes. This protein is involved in protein phosphorylation (GO:0006468), MAPK cascade (GO:0000165) and signal transduction (GO:0007165). Precisely, CAMK2B protein phosphorylates *STMN1* and *HSF1* gene products. Interestingly, STMN1 has been reported to be up-regulated during the progression of melanoma [22]. To date, STMN1 results also to be over-expressed across many human cancers, included cutaneous squamous cell carcinoma [23], a non melanoma skin cancer. This protein may as well represent an oncotarget as suggested by its down-regulation (−1.49), observed in proteomics analysis of MeWo cells treated with ascorbic acid. Finally, as can be noted in Figure 5C, SSBP1 network is not connected with ENO1, PTGES3, and NPM1 networks in SKCM. *SSBP1* encodes MtSSB (mitochondrial single-stranded DNA-binding protein). This gene is involved in trascriptional activaction of mitochondrial biogenesis (GO:0007005 as well as GO:0070584). The gene product takes part in DNA replication (GO:0006260) as a positive regulator of helicase activity (GO:0051096), and is also a subunit of a single-stranded DNA (ssDNA)-binding complex involved in the maintenance of genome stability [24]. The gene resulted quite altered (141/479 = 29%) in SKCM; its mRNA expression is regulated by GABPA and GABP1. Both genes are involved in the trascriptional activation of mitochondrial biogenesis (GO:0007005) and regulation of transcription from RNA polymerase II promoters (GO:0006357). It is interesting to note that GABPA and GABPB1 interact with YY1, which regulates MYC gene expression. MtSSB protein is phosphorylated by CHEK1 that plays a role in DNA damage checkpoint (GO:0000077), G2/M transition of mitotic cell cycle (GO:0000086) and DNA repair (GO:0006281). Here we found that MtSSB is upregulated in MeWo cells after ascorbic acid treatement (+ 1.77). Although it is difficult to envision its direct role as a therapeutic target, it should be highlighted that this protein protects p53 against ubiquitin dependent degradation. Furthermore SSBP1 associates with acetyltransferase p300 and promotes p53 acetylation [25]. SSBP1 upregulation by ascorbic acid could therefore be essential for an effective action of tumor suppressor p53.

In summary our findings demonstrate that an integrated approach involving proteomics, gene expression analysis and bioinformatics can be employed effectively to discover oncotargets. In particular we found that reduced cell viability and migration ability observed after AsA treatment in MeWo cells are associated to *ENO1*, *PTGES3*, *NPM1*, *PARK7* and *STMN1* genes dowregulation. Consistently, our data showed these genes to be overexpressed in melanoma cancer cells compared to normal melanocytes and dermal fibroblasts. The strong positive correlation between NMP1, *PTGES3*, PARK7 genes expression and migration ability suggests their involvement in the metastatic process. On the contrary, analysis of SSBP1 properties and its negative correlation with migration ability uncover an onco-suppressor gene in melanoma. In conclusion, the molecules described above, and in particular SSBP1 and PARK7, may be considered oncotargets suitable for the design of specific drugs or as specific molecular tools for the diagnosis and follow up in melanoma.

## MATERIALS AND METHODS

### Cells and AsA treatment

Two melanoma cell lines were used to assay the effects of AsA on gene and protein expression. A375 and Mewo melanoma cells (at passage 5 and 6 respectively, purchased from American Type Culture Collection (Rockville, MD, USA, no mycoplasm test was performed)) passage were cultured under humidified atmosphere of 5% CO_2_ and passaged in growth medium: DMEM/F12 containing 10% FBS (fetal bovine serum) supplemented with antibiotics (1% penicillin and streptomicyn) and 1% glutamin. Cells were then harvested using trypsin, washed and counted on a microscope using a Burker hemocytometer and plated again in growth medium. AsA was obtained from StemCell Technologies Inc. (Vancouver, BC, Canada) and dissolved in sterile water at a final concentration of 50 mM. Stock solutions were kept at −20°C for long-term storage and protected from exposure to light and air to prevent oxidation. Cells were also treated with ascorbate-2-phosphate (As2P) (Sigma-Aldrich; Merck Millipore) for a comparative evaluation.

Once 80% confluence was reached the cells were treated with ascorbic acid at concentrations ranging from to 0 to 1,000 μM .

### Cell viability

Cell viability was evaluated by the reduction of the tetrazolium salt XTT (sodium 3I-[1-phenylamino- carbonyl-3,4-tetrazolium]-bis(4-methoxy-6-nitro)benzene sulfonic acid hydrate- Cell proliferation kit II—XTT Roche) as previously reported [26].

In particular, 100 μl XTT labelling mixture were added to control and treated cells mantained at 37 °C in humidified atmosphere of 5% CO_2_ for 4 h. The spectrophotometric absorbance of the samples was evaluated by measuring the microtitre plate (ELISA) reader at a wavelength of 450 nm.

### 2DE proteomics analysis

2DE protein analysis from three biological replicates for a total of 1.5 × 10^6^ MeWo cells, untreated and treated with 500 μM AsA for 48 h, was performed as previously described [27]. Briefly, 500 μg of protein were subjected to IEF with 18 cm immobilized nonlinear pH 3–10 gradient IPG strips using a Protean IEF Cell (Bio-Rad). After IEF, IPG strips were equilibrated and then the proteins were separated using 8–18% SDS-PAGE gels. Ruthenium chelate (RuBPs) fluorescent staining was used to visualize protein spots on 2DE gel. Gels obtained by 2-DE were acquired using the VersaDoc (Bio-Rad Laboratories) and analyzed by PDQuest version 7.3 (Bio-Rad Laboratories), to obtain spot intensity quantification and gel matching. Normalized spot volumes were subjected to univariate and multivariate statistical analyses [28]. First of all an unsupervised Principal Component Analysis (PCA) was applied to detect global relationship among maps (Simca-P+ 13, Umetrics, Sweden). Then a Student’s t test (*p* < 0.01) was performed by using PDQuest, and multivariate statistics by using Simca-P+ 13 on log-transformed spot volumes. In particular, for identification of protein spots separating the two groups, we used orthogonal partial least squares discriminant analysis (OPLS-DA), applying a variable influence on projection (VIP) > 1.5 to identify the protein spots that most contributed to the calculated OPLS-DA model.

### Protein identification by nano-HPLC-Chip ion trap mass spectrometry

Spots were selected for identification based on significant differences in Student’t *t*-test (*p* < 0.01) and/or good correlations [p(corr) > 0.75] by the multivariate OPLS-DA. Protein identification was performed after in-gel trypsin digestion, as previously described [29]. Briefly, peptides from each sample were separated by RP nano-HPLC-Chip technology (Agilent Technologies, Palo Alto, CA, USA) online-coupled with a 3D ion trap mass spectrometer (model Esquire 6000, Bruker Daltonics, Bremen, Germany). Database searches were conducted using the MS/MS ion search of Mascot against human entries of the non-redundant NCBI database.

### Protein annotation and proteins interactions

Functional annotation of identified proteins was performed according to Gene Ontology (GO) using the PANTHER classification system v 9.0 (http://www.pantherdb.org/) according to cellular components, molecular functions and biological processes. Protein interactions were assessed using the STRING database (http://string-db.org). In addition, STRING analysis was performed to reconstruct the functional association of ENO1, PARK7, PTGES3, NPM1, SSBP1, STMN1 among them and other proteins involved in apoptosis and metastasis, by querying genes involved in the progression and metastatic potential of melanoma reported by Riker [30]. We retrieved interactions that were of at least medium confidence (score 0.4) based exclusively on coexpression, experimental and database knowledge while excluding all other prediction methods implemented in STRING (such as textmining and gene fusion). Network depth was kept to the minimum value (1) and no additional white nodes were added in order to exclude as many false positive interactions as possible.

### Cell cultures

A total of 5 malignant melanoma lines (A375 (at passage 5)), MeWo ((at passage 6)), MEL-HO (at passage 5), Colo-800 (at passage 4) and Colo-853 (at passage 5)) plus a melanocyte cell line (NHEM, at passage 2) were purchased from American Type Culture Collection (Rockville, MD, USA); a dermal fibroblast culture was obtained from a skin biopsy of a normal individual (control) after appropriate consent. No mycoplasm test was performed. Cell lines were cultured in RPMI 1640 or DMEM (Sigma-Aldrich; Merck Millipore, Darmstadt, Germany) with 10% foetal bovine serum (FBS) (Sigma-Aldrich; Merck Millipore) according to manufacturer’s instructions. Adherent cells for each cell line were harvested to perform molecular and cellular analyses. For each cell line, three different cultures were tested.

### Total RNA extraction

Total RNA was extracted by using the RNAeasy minikit (Quiagen) with DNAse I and analyzed by measuring the absorbance at 260 nm and 280 nm. RNA integrity was confirmed by RNA electrophoresis on a 1.5% agarose gel containing ethidium bromide.

### Reverse transcription

First-strand cDNA was obtained by using the First Strand cDNA Synthesis Kit (GE Healthcare), with random hexamers, (GE Healthcare) according to the manufacturer’s protocol. RT products were stored at −80°C.

### Real time RT-PCR

PCRs were performed in a total volume of 25 μl containing PCR Master with ROX premixed with SYBR Green and 20 ng of cDNA from each sample The following primer sets were used: *ENO1(FW TAACAACAACCTGAAGAATG -RV-TCCAGGCCTTCTTTATTCTC) STMN1 (FW-AGATGT ACTTCTGGACTCAC-RV GATCAGACCAGGTA ATCAATG)-NMP1 (FW-GCCAGTGAAGAAATCTATACG-RV TACAGAACTAGGTCCTTTTGG)–(SSBP1 FW-GGTG ATGTCAGTCAAAAGAC- RV-TCACATATTGATATGCC ACG) - PTGES3 (FW CAGATGATGATTCACAAGACAG-RV -CTTTAGAGCTATCAACTCAGG)-PARK7 (FW-CTGT TGGCTCATGAAATAGG-RV-GTGTAATGACCTCCATTC ATC)-*

Real Time RT-PCR reactions were carried out in two-tube system and in multiplex. The Real Time amplifications included 10 minutes at 95°C (AmpliTaq Gold activation), followed by 40 cycles at 95°C for 15 seconds and at 60°C for 1 minute. Thermocycling and signal detection were performed with ABI Prism 7300 Sequence Detector; (Applied Biosystems). Signals were detected according to the manufacturer’s instructions. Gene expression was calculated after normalization against the housekeeping genes (B2M, GAPDH) using the relative fold expression differences. Ct values for each reaction were determined using TaqMan SDS analysis software. For each amount of RNA tested triplicate Ct values were averaged.

### RNA interference

We analysed the effects of NMP1 and PARK7 genes knockdown by using MISSION shRNA specific for NMP1 (SequenceA: CCGGGCCAAGAATGTGTTGTCC AAACTCGAGTTTGG ACAACACATTCTTGGCTTT TTG;SequenceB: CCGGGCGCCAGTGAAGAAATCTAT AC TCGAGTATAGATTTCTTCACTGGCGCTTT TTG;SequenceC: CCGGGCCGACAAAGAT TATCACTTTCTCGAGAAAGTGATAATCTTTGTC GGCTTTTTG;SequenceD:CCGGCCTAGTTCTGTAGA AGACATTCTCGAGAATGTCTTCTACAGAACT AGGTTTTTG; PARK7 (SequenceA:CCGGACTCTGAGAATCGTGTGGAAACTCGAGTTTCCACACGATTCT CAGAGTTTTTT;SequenceB: CCGGGCTGGGATTAA GGTCACCGTTCTCGAGAACGGTG ACCTTAATCCCAGCTTTTT;SequenceC:CCGGGTAGCCGT GATGTGGTCATTTCTCGAGAAATGACCACATCA CGGCTACTTTTT;Sequence D: CCGGGCAATTGTT GAAGCCCT GAATCTCGAGATTCAGGGCTTC AACAATTGCTTTTT) and non-targeting controls (control and scrambled) (from Sigma-Aldrich (St. Louis, MO)) as previously reported [31]. In the following experiments, the four sequences for each target were applied combined two at a time (shRNA-A and shRNA-B) as previously described [31]. Briefly, 1.6 × 10^4^ cells were seeded at low density into 96- well plates. After 12 h (cell confluency of 70%) shRNA Lentiviral Particles supplemented with 8 μg/ml hexadimethrine bromide was added and then replaced after 8 h with normal growth medium.

### Migration ability

A scratch test was used in order to evaluate migration ability. The cells were cultured in 48-well plates and grown to 80% confluence. The scratch track was produced by using a 200-μl pipette tip. The supernatants were removed and the plates were washed with PBS and grown in fresh medium. Cell migration was observed in timelapse (EVOS) and the relative migration was calculated as previously reported [32], i.e. as relative migration distance (RMD) (%) = 100 (A–B)/A, with A and B representing the width of cell scratches before and after incubation, respectively. Images were in 3 independent experiments.

### Cbio genomics cancer portal analyses

To investigate the involvement of *ENO1*, *PARK7*, *PTGES3*, *NPM1*, *SSBP1* and *STMN1* in the Skin Cutaneous Melanoma (SKCM), we carried out a preliminary analysis on these genes by means of the open-resource cBio Cancer Genomics Portal (http://www.cbioportal.org/), using the provisional data set about the SKCM (479 samples) stored in the Cancer Genome Atlas (TCGA; https://cancergenome.nih.gov/). We analyzed the six genes for different genomic profilings such as alterations (i.e., amplification, deep deletion, mRNA and protein expression changes, and missense mutations), mRNA expression (RNA Seq V2 RSEM), and Protein expression (*Reverse phase protein array,* RPPA). To analyze mRNA and protein expression, we used the default z-score threshold ± 2 both RNA Seq V2 RSEM and RPPA, respectively. Furthermore, we investigated whether events involving such queried genes happen in a way of mutual exclusivity or co-occurrence in order to contribute to development and progression of SKCM. The enrichment analysis was used to find mRNA expression changes and, if available, protein expression changes. We also took into account the expression correlation among them.

Finally, to locate the interacting genes and the pathways engaging the query genes in SKCM, we looked for the network involving *ENO1*, *PARK7*, *PTGES3*, *NPM1*, *SSBP1* and *STMN1*. In fact, the cBio Portal offers the possibility to generate automatically networks containing the query genes and their alterated neighbour-genes in the type of investigated cancer. Usually, the generated network shows only 50 neighbour-genes with the highest genomic alteration frequency in addition to the query genes. This entails the advantage of managing network complexity and automatically drawing attention to the most relevant genes, thanks to the visualization of networks which are altered into the cancer type of interest.

### Statistical analysis

Results were expressed as mean ± S.E. Statistical analysis was assessed by one-way analysis of variance (ANOVA). Differences between groups yielding a statistical significance with *p* < 0.05 were tested with Bonferroni as a post hoc test. Analyses were applied to experiments carried out at least three times. Statistical analyses were performed using SPSS for Windows, version 16.0 (SPSS Inc, Chicago, IL, USA).

## SUPPLEMENTARY MATERIALS FIGURES AND TABLES










