# Supplementary material for: An integrated approach identifies new oncotargets in melanoma
Source: Oncotarget. 2017 Dec 15;9(14):11489–502. doi: 10.18632/oncotarget.23727 (PMC5837771; doi:10.18632/oncotarget.23727)
Supplement: Supplementary file 1 [file oncotarget-09-11489-s001.pdf]

An integrated approach identifies new oncotargets in melanoma

SUPPLEMENTARY MATERIALS

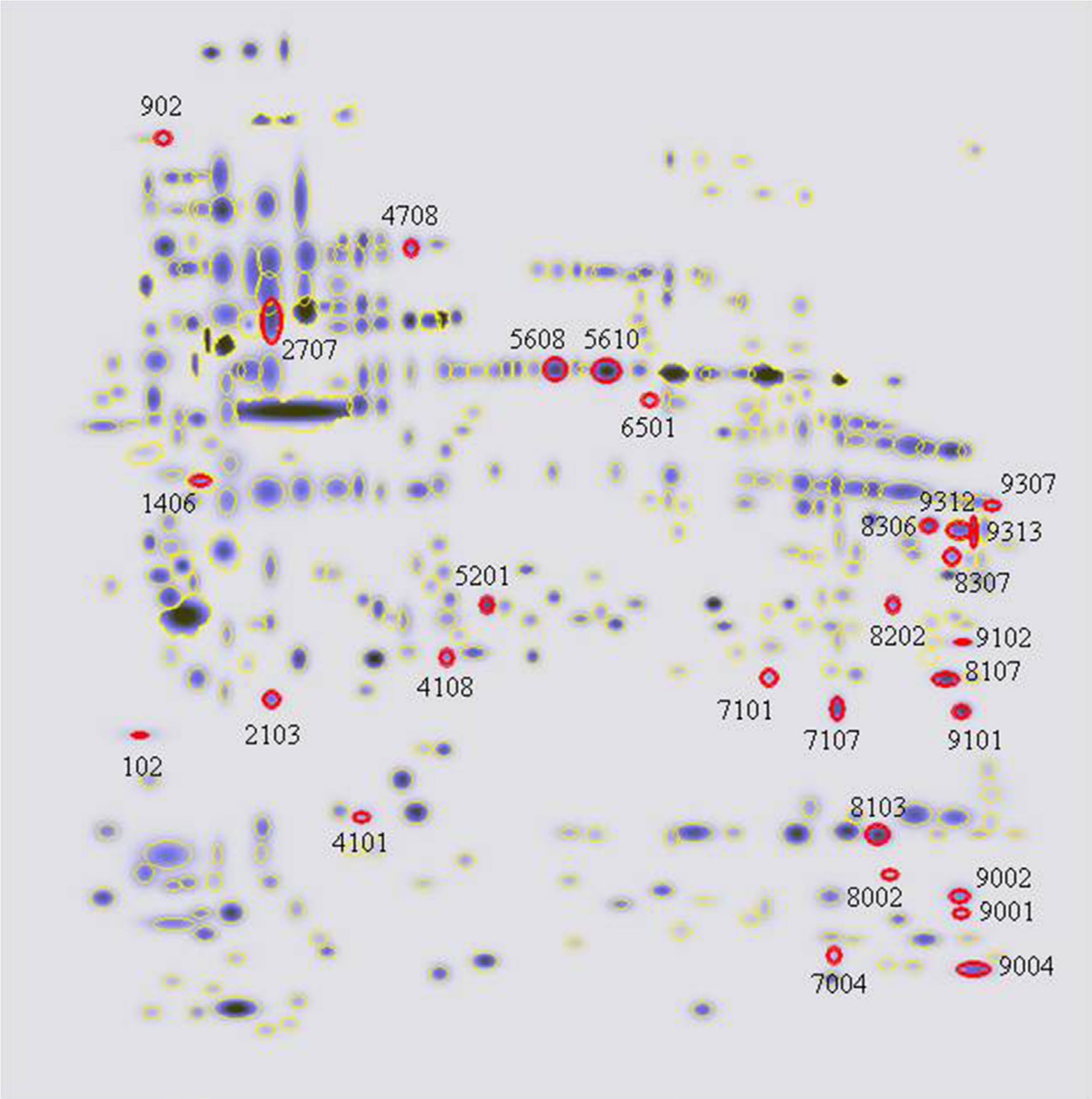

Supplementary Figure 1: Identified protein species marked with the numbers corresponding to the standard spot number.

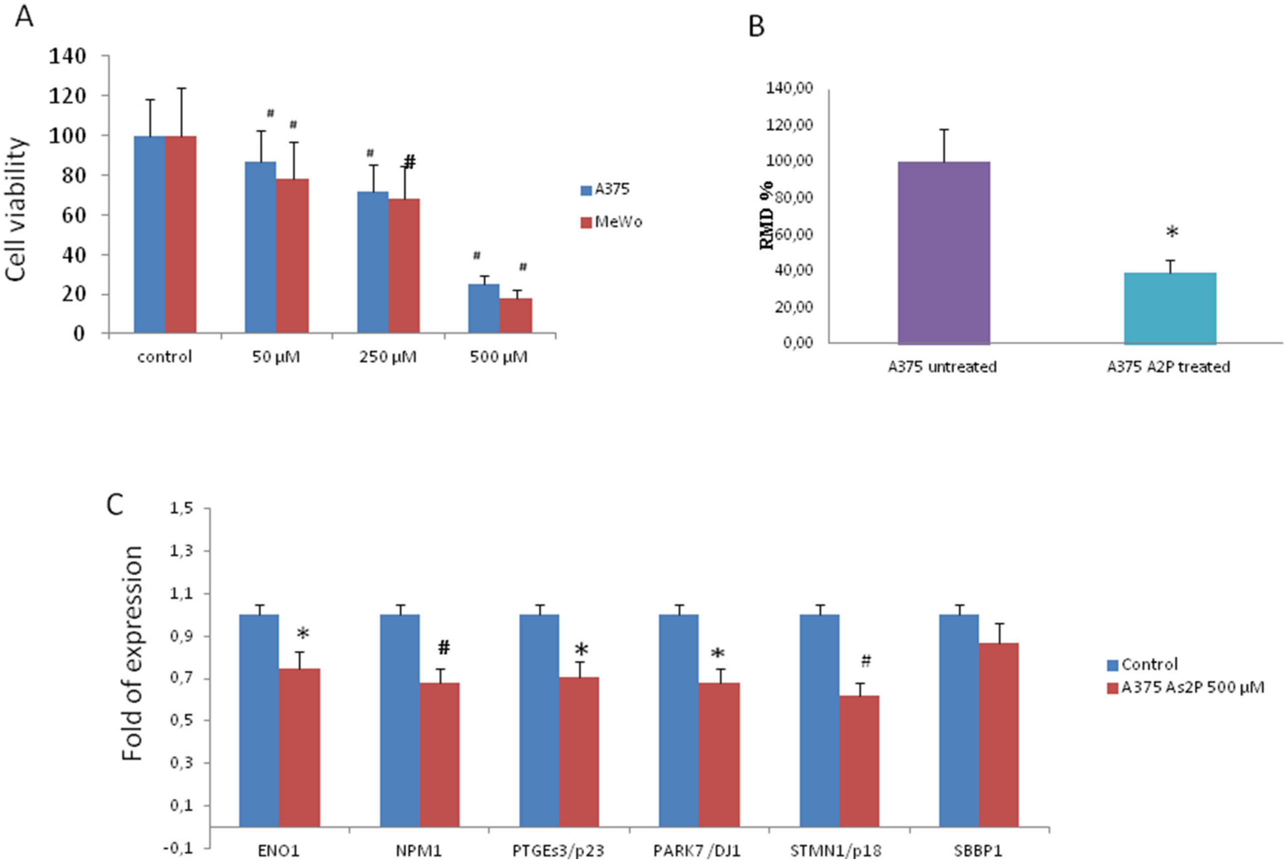

Supplementary Figure 2: Cell viability in Mewo and A375A (**A**), migration ability of A375 cells (**B**) and gene expression modulation in A375 in A2P treated cells. ($*p < 0.05$; $^{\#}p < 0.01$).

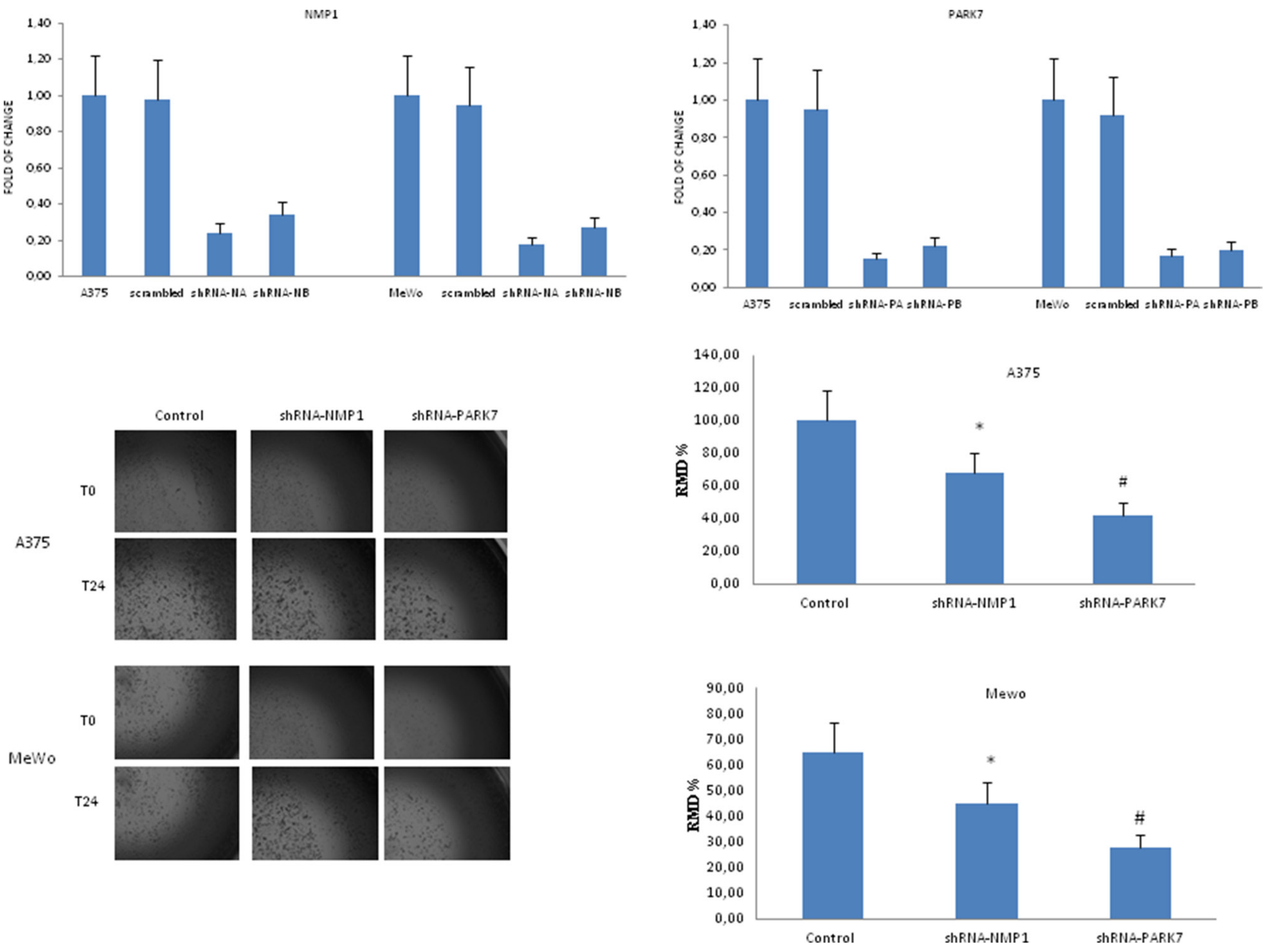

Supplemental Figure 3: A375 and MeWo melanoma cells were transduced by lentiviral particles encoding sequences for different shRNAs specific for NMP1 (shRNA-NA and shRNA-NB) and PARK7 (shRNA-PA and shRNA-PB) and negative control (scrambled). Transduction efficiency was monitored after 24 h by Real Time gene expression analysis (**A**). Migration ability in knockdown NMP1 and PARK7 cells was evaluated by Scratch test (**B**) and Relative Migration Distance values (**C**) *($p < 0.05$), #($p < 0.01$).

Supplementary Table 1: Proteins identified and apoptosis. See Supplementary_Table_1

Supplementary Table 2: Protein-protein functional association in apoptosis. See Supplementary_Table_2

Supplementary Table 3: Proteins identified and metastasis. See Supplementary_Table_3

Supplementary Table 4: Protein-protein functional association in metastasis. See Supplementary_Table_4

Supplementary Table 5: Mutual exclusion and co-occurrence analysis

Gene Pairs		co-occurrence			
**Gene A**	**Gene B**	***p*-Value**	**Log Odds Ratio**	**Association**	**Statistical significant**
ENO1	***PTGES3***	0.00000229	1.953	Tendency towards co-occurrence	**Significant**
PARK7	***STMN1***	0.0000101	1.607	Tendency towards co-occurrence	**Significant**
ENO1	***PARK7***	0.0000568	1.587	Tendency towards co-occurrence	**Significant**
NPM1	***PARK7***	0.0183	1.1678	Tendency towards co-occurrence	**Significant**
PARK7	***SSBP1***	0.000454	1.009	Tendency towards co-occurrence	**Significant**
PTGES3	***SSBP1***	0.00555	0.874	Tendency towards co-occurrence	**Significant**
ENO1	*NPM1*	0.0526	1.065	Tendency towards co-occurrence	
ENO1	*STMN1*	0.0561	0.800	Tendency towards co-occurrence	
PTGES3	*NPM1*	0.0683	0.978	Tendency towards co-occurrence	
NPM1	*STMN1*	0.114	0.796	Tendency towards co-occurrence	
PTGES3	*PARK7*	0.125	0.583	Tendency towards co-occurrence	
NPM1	*SSBP1*	0.166	0.497	Tendency towards co-occurrence	
STMN1	*SSBP1*	0.308	0.203	Tendency towards co-occurrence	
**Gene Pairs**				**mutual exclusion**	
**Gene A**	**Gene B**	***p*-Value**	**Log Odds Ratio**	**Association**	**Statistical significant**
ENO1	*SSBP1*	0.427	-0.141	Tendency towards mutual exclusion	
PTGES3	*STMN1*	0.126	-0.980	Tendency towards mutual exclusion	
